# Carotid Stent Fracture and Stent Fragment Migration in a Patient with Eagle Syndrome

**DOI:** 10.1007/s00062-025-01571-y

**Published:** 2025-09-22

**Authors:** Alexander Brose, Omar Al-Qaisi, Anne Mrochen, Christian Claudi, Christoph Arens, Christine Langer, Tobias Struffert

**Affiliations:** 1 Department of Neuroradiology, University Hospital Giessen, Justus-Liebig-University Giessen, Klinikstraße 33, 35392 Giessen, Germany; 2 Department of Neurology, University Hospital Giessen, Justus-Liebig-University, Giessen, Germany; 3 Department of Otorhinolaryngology, University Hospital Giessen, Justus-Liebig-University, Giessen, Germany

## Introduction

Vascular compression by an elongated styloid process (Eagle Syndrome) was first described by the American otolaryngologist Watt Eagle [1, 2] in 1937. It was initially defined as a clinical entity of an elongated (> 2.5 cm) or abnormally shaped styloid process (SP) causing headaches or facial pain by compression of the internal (ICA) or external carotid artery (ECA)—in addition to the “classical” neuropathic variant causing odynophagia and the persistent feeling of a sore throat after tonsillectomy [3]. Other symptoms include dysphagia, tinnitus, and even bleeding [4, 5]. In the past decades, Eagle syndrome gained attention as a possible explanation of dissections or pseudoaneurysms of the cervical ICA [5, 6]. Along with this in patients with dissections treated by stents it has been reported that stent fractures due to compression by the SP [6–10] may occur. We describe a case of spontaneous ICA dissection due to vascular Eagle syndrome treated as an emergency by carotid artery stenting (CAS), with subsequent stent fracture and fragment migration in the long term.

## Case Description

**Fig. 1:**
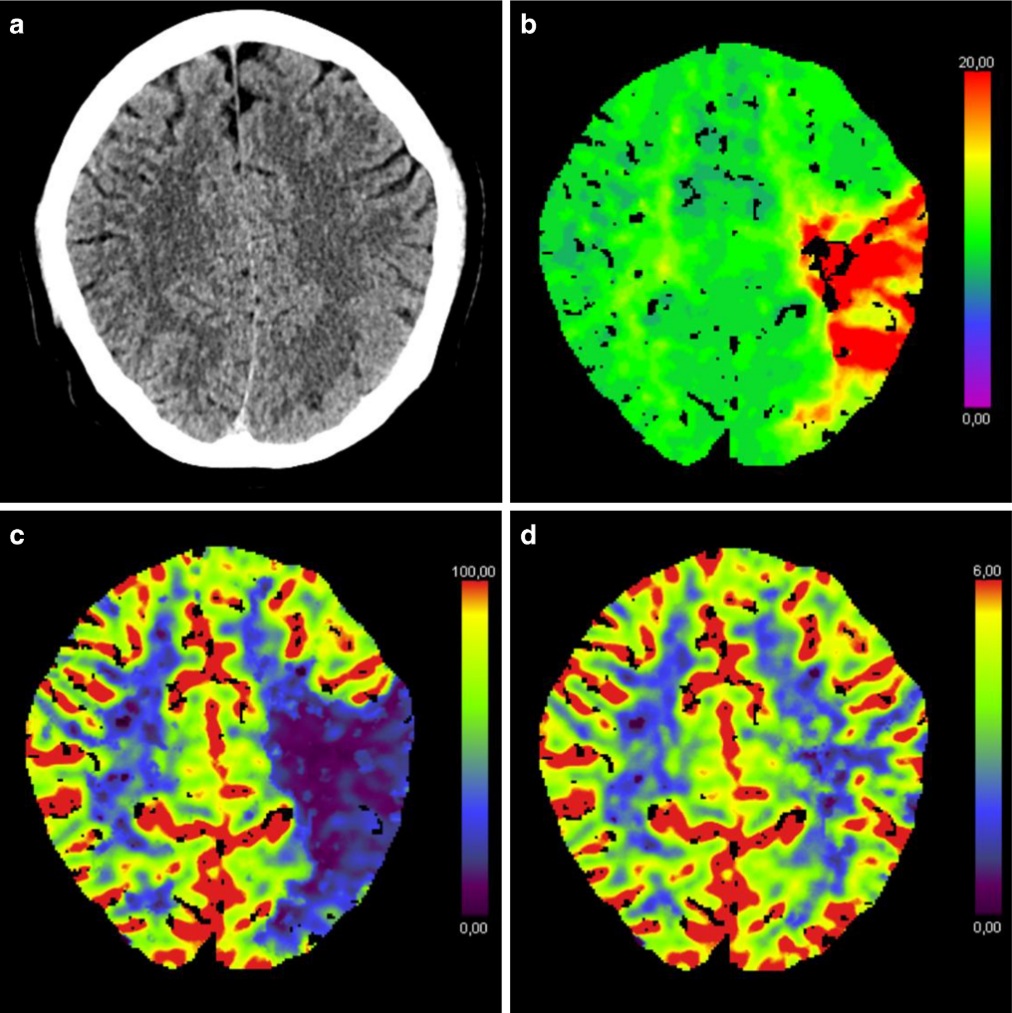
Unenhanced brain CT scan (**a**) shows intact corticomedullary differentiation. Contrast enhanced CT perfusion study (**b**, **c**, **d**) reveals elevated time to peak (**b**), impaired cerebral blood flow (**c**), and preserved cerebral blood volume (**d**) of the left frontal and parietal lobes including the central region

**Fig. 2:**
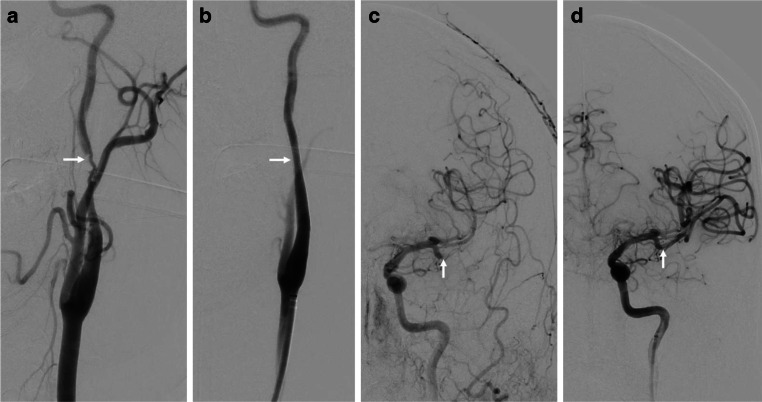
Digital subtraction angiography (DSA) of the cervical segment of the left internal carotid artery before and after carotid artery stenting (**a**, **b**) shows an irregular contrast filling of the dissected vessel lumen (white arrow in **a**) and smooth vessel contours after stenting (white arrow in **b**). DSA of the intracranial segment of the left internal carotid artery before and after mechanical recanalization (**c**, **d**) shows an occlusion of the inferior trunk at the M2 level (white arrow in **c**) and perfect treatment success after mechanical recanalization (white arrow in **d**, TICI grade 3)

**Fig. 3:**
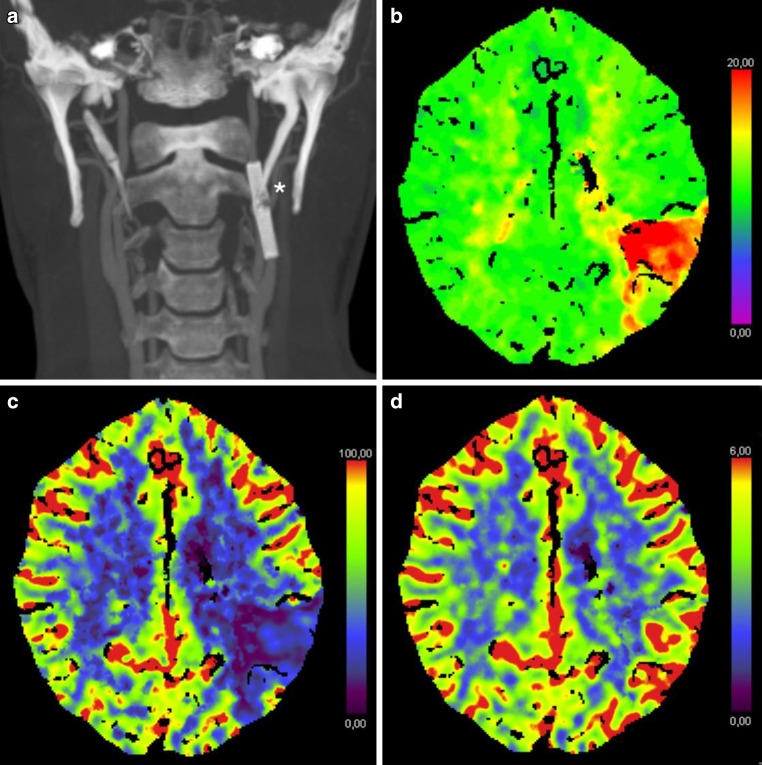
Coronal maximum intensity projection (MIP) of the CT angiography scan (**a**) depicts the fractured ICA stent in close vicinity to an elongated and thickend left styloid process (white asterix in **a**). Contrast enhanced CT perfusion study (**b**, **c**, **d**) reveals elevated time to peak (**b**), impaired cerebral blood flow (**c**), and preserved cerebral blood volume (**d**) of the left postcentral region in the posterior vessel territory of the left MCA

**Fig. 4:**
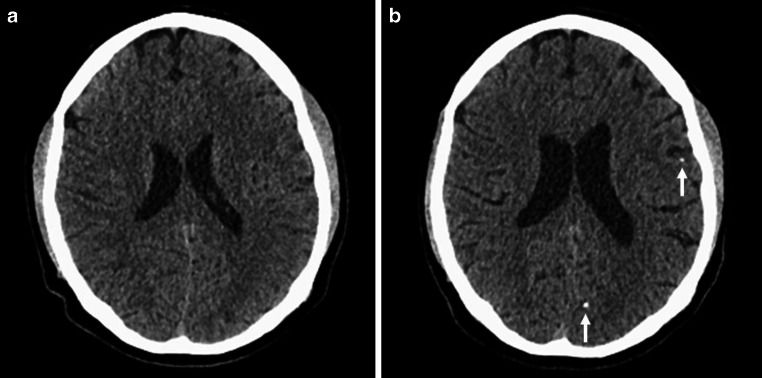
Unenhanced brain CT scan at discharge after the first treatment (**a**) and on admission for the second stroke (**b**). CT scan after carotid artery stenting and stent fracture shows hyperdense stent fragments in the left frontal lobe and the parietooccipital fissure via fetal origin of the posterior cerebral artery (white arrows in **b**) which were not present before stenting (**a**)

**Fig. 5:**
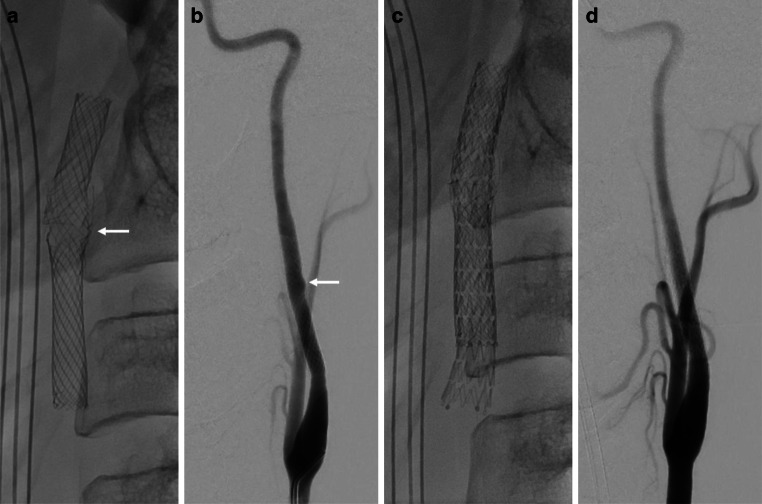
Flat panel detector computed radiography in oblique view (**a**, **c**) and digital subtraction angiography (**b**, **d**) of the cervical segment of the left ICA before (**a**, **b**) and after (**c**, **d**) re-stenting. A fracture of the middle part of the stent (Carotid Wallstent) is evident with subsequent damage to the mesh architecture (white arrow in **a**) and a flat-based outpouching of the vessel lumen (white arrow in **b**). Resulting stent-in-stent architecture (**c**) with homogenously deployed new stent (CGuard) and smooth vessel contour of the left ICA (**d**). Please note that the SP is not visible in (**a**)

A 45-year-old, previously healthy, male patient presented to our hospital’s emergency department with acute onset of left hemispheric stroke including aphasia, hemiparesis and gaze deviation. Computed tomography (CT) with CT angiography (CTA) revealed an irregular, most likely dissected lumen of the left ICA and occlusion of the left middle cerebral artery (MCA). CT perfusion imaging showed involvement of the left central region (Fig. 1). Digital subtraction angiography (DSA) confirmed the findings and the patient was consecutively treated by emergency stenting of the ICA (Boston Scientific Carotid Wallstent, 7 × 30 mm) and successful mechanical thrombectomy of the left MCA (Fig. 2). The patient’s clinical condition improved immediately post treatment. Due to clinical deterioration a few hours later, magnetic resonance imaging (MRI) was performed and revealed a small parenchymal haemorrhage of the left basal frontal lobe. This region was not involved in the stroke region and the bleeding was attributed to concurrent systemic thrombolysis. Despite this, the patient recovered very well and returned back to work two months later. Follow up brain MRI and ultrasound examination for stent patency at 6 months were unremarkable. By then, the patient was kept on lifelong Aspirin medication after initial dual antiplatelet therapy. Twelve months after this incident, the patient was admitted again as an emergency by acute onset of hemiparesis and aphasia. CTA showed fracture of the carotid stent followed by a distal MCA occlusion at the M3 level. CT perfusion study confirmed involvement of the left central region explaining the severe clinical condition. In this CTA scan the close proximity to an elongated and thickened styloid process (SP) was recognized (Fig. 3), which was present in the initial CTA as well when reviewed in retrospect. Unenhanced CT of the brain showed multiple distally migrated stent fragments, which were new compared to prior examinations (Fig. 4). The patient received systemic thrombolytic therapy and was referred to the angio suite where the stent fracture could be confirmed as well as a flat-based outpouching of the vessel lumen. Mechanical thrombectomy (aspiration) and intra-arterial thrombolysis therapy due to M3 occlusion was performed and recanalization could be achieved. Due to prior haemorrhagic complication, a new stent was not implanted immediately. The patient’s clinical situation improved following emergency treatment. But due to clinical deterioration 8 h later, MRI was performed and haemorrhage of the acute affected brain tissue was noted. No specific action was taken on this, the patient’s clinical condition was stabilized. Since Eagle syndrome had now been confirmed, elective surgical resection of the elongated left styloid process has been performed. Resection of the styloid process was performed transoral after tonsillectomy. The styloid process was located endoscope assisted via the parapharyngeal surgical approach and severed at the skull base. Bone protrusions were smoothed with a diamond burr. After complete healing of the parapharyngeal approach, re-stenting of the left ICA has been performed six weeks later in typical fashion (Fig. 5). We choose to implant a stent with closed-cell design and a small mesh pore size (InspireMD CGuard, 8 × 40 mm) to reduce the risk of plaque protrusion. Six months later, the patient still suffered from mild aphasia and hemiparesis.

## Discussion

The normal length of the SP is less than 2.5 cm, and its tip is located between the ICA and ECA, lateral to the tonsillar fossa [1]. In the general population, the prevalence of an elongated SP of more than 3 cm is approximately 4–10%, but symptoms occur in only 4% of these individuals [2, 5, 10]. There may be a lack of precise epidemiological knowledge about the incidence in the population and the frequency of symptoms. This entity is probably underdiagnosed. We could not identify any information about the likelihood of a dissection in patients suffering from Eagle syndrome. In a systematic literature review *Pagano et al.* identified 37 cases of internal carotid artery dissection (CAD) caused by Eagle syndrome [5]. CAD was discovered incidentally in only one case, while all other cases described an acute onset, with hemiparesis being the most common symptom in one-fifth of the patients. Other common clinical features included aphasia, loss of consciousness, amaurosis, and headache [5]. Despite its benign nature, Eagle syndrome can lead to severe complications including reports on transient ischemic attacks and strokes of the downstream territories of ICA [5–7, 11]. An elongated SP has been recognized as a risk factor for CAD, although many of the reported cases also describe an asymptomatic elongated SP on the unaffected contralateral side [5, 10]. The mechanisms underlying vascular Eagle Syndrome and its association with carotid artery dissection and potential stent fractures remain poorly understood. In our case, we assume that direct compression of the first stent by the elongated SP was the reason for the stent fracture. To the best of our knowledge, there are only five additional reported cases of stent fractures due to Eagle’s Syndrome in current literature (Table 1). Hooker *et al.* were one of the first groups to report on a stent fracture of an Xact carotid artery stent (Abbott) due to an elongated SP [7]. Their patient previously underwent emergent CAS due to severe ICA stenosis and stroke. CTA at the one-year follow-up appointment showed a fractured stent and complete ICA occlusion. Since the patient did not develop new symptoms, no immediate intervention was performed [7]. A similar case of fracture of a Carotid Wallstent (Boston Scientific) was reported by Yano *et al.* in a patient who underwent emergent CAS of ICA dissection due to Eagle Syndrome [6]. Due to the subsequent aneurysm formation at the site of the fracture, coil embolization and re-stenting were performed in this case, followed by the surgical resection of the elongated SP. The group also reviewed 20 case reports of carotid artery dissections due to Eagle syndrome and suspected an increased incidence of spontaneous ACI dissection in patients with Eagle Syndrome [6]. Tan *et al.* described the deformation of a CASPER carotid artery stent (MicroVention, Terumo) in a patient with ongoing headache and neck pain two weeks after stent implantation [8]. Although complete fracture of the stent did not occur in this case, long time outcome was not yet reported. Pfaff *et al.* described the fracture of a FRED flow diverter (MicroVention, Terumo) in a patient who was treated for dissection and pseudoaneurysm formation of the left ICA [9]. In this case, the fracture of the flow diverter ultimately led to complete occlusion of the left extracranial ICA 8 months after implantation without any symptoms. No interventional treatment was initiated. Nishihori *et al.* reported on the postoperative fragmentation and distal migration of a carotid artery stent one year after coil embolization and stenting of a left cervical internal carotid artery aneurysm [10]. The patient subsequently underwent resection of the extended SP followed by repeat CAS. And, recently, Ion *et al.* commented on a case of carotid artery stent fracture after extreme neck extension, which the group attributed to a compression by a calcified thyroid cartilage [12], similar to the cases of vascular Eagle syndrome. Unfortunately, in the latter two cases the stent types were not specified.

**Table 1:** Cases of cervical internal carotid artery stent fractures attributed to Eagle Syndrome.

	Stent type	Time after stent implantation	Symptoms
Hooker *et al.* [7]	Xact (6–8 × 40 mm)	1 year	Asymptomatic
Yano *et al. *[6]	WALLSTENT (8 × 28 mm)	3 months	Asymptomatic
Tan et al. [8]	CASPER (7 × 18 mm)	2 weeks	Headache, neck pain
Pfaff *et al.* [9]	FRED (5.5 × 32/26 mm)	3 months	Asymptomatic
Nishihori *et al.* [10]	Not specified	1 year	Asymptomatic

In our case the presence of Eagle syndrome had not been recognized upon first admission. Its significance was unfortunately not noticed until the second admission. We must assume, that the dissection as well as the stent fracture occurred most likely due to Eagle syndrome attributed to the patient’s elongated SP. In addition to the stent fracture, we also observed distal migration of stent debris to the brain. Fractured stents can obviously fragment in such way that stent debris can enter the cerebral vessels. It seems that our case is one of four cases of stent debris migration in literature that have been published so far [10, 12, 13]. The dispersed stent fragments were found to be asymptomatic in the cases described by Nishihori *et al. *and Silva *et al. *[10, 13]. In contrast, *Ion et al. *were able to observe the simultaneous occurrence of debris and multifocal cerebral infarction in the right middle cerebral artery territory after stent fracture of the right ICA [12]. In our case, debris was not present at the occluded M3 vessel. We are convinced that the fractured stent caused the second stroke and assume clot formation at the site of the stent fracture and consecutive distal migration of emboli. Following this hypothesis, we implanted a second stent after surgical resection of the elongated SP. Due to the small number of cases and unclear epidemiology, the appropriate treatment strategy for carotid stenting in Eagle Syndrome remains controversial [5, 10]. Possible approaches consist of stenting in combination with surgical resection of the SP [6, 7, 9]. The styloidectomy seems to be necessary to prevent further damage to the implanted stent [5]. In our case, an interdisciplinary treatment approach seemed reasonable and the patient underwent surgical styloidectomy and repeated CAS in combination with concomitant double antiplatelet therapy.

## Conclusion

Vascular Eagle Syndrome should be considered as a possible cause of spontaneous ICA dissection, especially in young patients. CT angiography should be carefully evaluated for an elongated or abnormally shaped styloid process which may lead to stent fractures after carotid artery stenting and subsequent complications like thromboembolic events. The exact epidemiology and the risk for dissection and carotid stent fractures due to Eagle Syndrome remain unclear.

The described treatment process appears to be mandatory but is not proven due to a lack of epidemiological data. Surgical resection of the styloid process may be the treatment of choice to prevent stent fractures.

It seems that the Carotid Wallstent could release fragments which migrate to distal cerebral vessels. The significance and potential treatment of this migrated debris remain unclear.
